# Spatial sugar separation is key to how fast you get old

**DOI:** 10.1093/plphys/kiae494

**Published:** 2024-09-17

**Authors:** Ryo Yokoyama

**Affiliations:** Plant Physiology, American Society of Plant Biologists; Max-Planck-Institute of Molecular Plant Physiology, Am Mühlenberg 1, Potsdam-Golm 14476, Germany

In eukaryotic cells, biological membranes separate metabolites. This metabolite compartmentalization fulfills 3 functions in metabolism (Fig. A): (1) to provide unique chemical and biophysical environments (e.g. pH and redox) for efficient biochemical reactions and metabolite storage; (2) to protect intracellular components from toxic intermediates and by-products; and (3) to facilitate the metabolic control that allows to regulate metabolite storage and release, prevent futile cycles, and provide a mechanism by which metabolites function directly as signaling molecules (Bar-Peled and Kory 2022). Transporters play an important role in actively translocating metabolites to regulate metabolite-mediated biological functions. The discovery of novel transporters and their substrates brings us breakthroughs in understanding molecular mechanisms of metabolite compartmentalization that affects various cellular functions.

**Figure.**
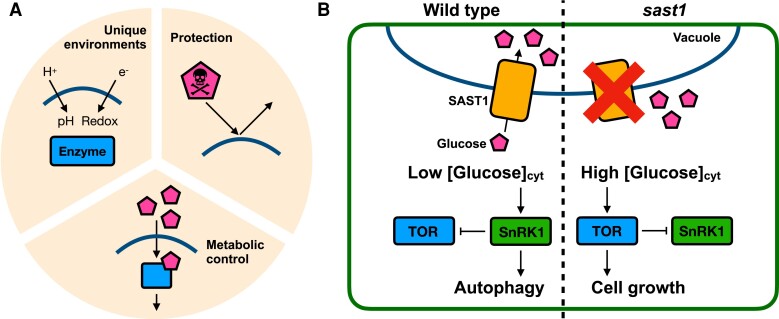
**A)** The 3 major cellular roles of metabolite compartmentalization: (1) generation of unique chemical and biophysical conditions that contribute to efficient biochemical reaction and metabolite storage; (2) protection of cellular components from toxic compounds; and (3) facilitation of metabolite control that enables efficient signaling, prevents futile cycles, and allows metabolite storage and release. **B)** A model of the SAST1 function during senescence. The concept of Figures A and B originated from Bar-Peled et al. and Cheng et al., respectively.

In this issue of *Plant Physiology*, Cheng et al. identified a novel sugar transporter that mediates sugar homeostasis during the leaf senescence of *Arabidopsis thaliana* (Cheng et al. 2024). Senescence is a process of growing old, during which multiple signaling cascades trigger the breakdown of cellular compartments and metabolites for metabolic remodeling that helps to redistribute essential nutrition (e.g. nitrogen) at an intracellular level (Woo et al. 2019). Key players in the early stage of senescence are 2 signaling pathways mediated by TARGET OF RAPAMYCIN (TOR) and SUCROSE-NON-FERMENTING-1-RELATED PROTEIN KINASE 1 (SnRK1) (Artins et al. 2024). TOR is a master regulator that promotes growth and maintains intracellular metabolic activity under carbon-sufficient conditions. SnRK1 acts as an antagonist to TOR to adjust the downstream metabolic cascades, including autophagy, under low cellular carbon energy status. Previous studies revealed that TOR and SnRK1 monitored the cytosolic glucose level (Artins et al. 2024). However, how the cytosolic glucose concentration is regulated under various developmental conditions, including senescence, remains to be elucidated.

Cheng et al. focused on a poorly characterized Arabidopsis gene encoding the Sugar-porter Family Protein 1 (SFP1), the expression of which was reportedly upregulated during leaf senescence (Quirino et al. 2001). Consistent with this previous study, SFP1 expression was upregulated by both natural developmental senescence and artificially induced senescence by abscisic acid treatment. SFP1 was localized to the vacuolar membrane (tonoplast) and transported glucose in vitro. To address its in planta role in plant physiology, the authors isolated its knockout mutants and demonstrated that they exhibited delayed senescence (stay-green) phenotype, leading to the renaming of SFP1 to Senescence-Associated Sugar Transporter1 (SAST1). Non-aqueous fractionation (NAF) is a method that can measure the subcellular metabolite distribution in plant cells by fractionating intact subcellular compartments (Medeiros et al. 2019). Using this technique, it was revealed that, under the senescent conditions, the cytosolic glucose level in the *sast1* mutants was up to 14-fold higher than in the wild-type fraction. During the senescence, the *sast1* mutants showed the upregulation and downregulation of TOR and SnRK1 activities, resulting in the impacts on various downstream cascades that included slowing down autophagy-dependent senescence. Taken together, these results led the authors to propose a model where *SAST1* expression is upregulated during senescence to accelerate glucose import from cytosol to vacuole, inducing autophagy-related senescence by the TOR-SnRK1–dependent signaling cascade under low cytosolic glucose conditions (Fig. B).

This study provides a perfect example of the third function of metabolite compartmentalization, highlighting the key role of STAT1-mediated glucose compartmentalization in governing the downstream signaling network. One important lesson from the work is how effective the NAF approach is in analyzing metabolite distributions (Medeiros et al. 2019). To further examine dynamic aspects of metabolite compartmentalization, new technologies recently have been advancing rapidly. For example, matrix-assisted laser desorption-ionization mass spectrometry imaging can map metabolite distributions on biological samples in a quantitative manner (Liu et al. 2022). Many genetically encoded metabolite sensors have been developed to enable real-time observation of metabolite levels at the subcellular level (Choe and Titov 2022). Along with such technological advances, the roles of metabolite compartmentalization will be further addressed in various biological phenomena.

## Data Availability

This manuscript contains no experimental data.
